# Assessing Feasibility and Barriers to Implementing a Family-Based Intervention in Opioid Treatment Programs

**DOI:** 10.1007/s11414-023-09873-0

**Published:** 2023-12-14

**Authors:** Khary K. Rigg, Steven L. Proctor, Ethan S. Kusiak, Sharon A. Barber, Lara W. Asous, Tyler S. Bartholomew

**Affiliations:** 1 Department of Mental Health Law & Policy, University of South Florida, 13301 Bruce B. Downs Blvd, Tampa, FL 33612 USA; 2 Thriving Minds of South Florida, Miami, FL 33126 USA; 3 Department of Public Health Sciences, University of Miami Miller School of Medicine, Miami, FL USA

## Abstract

Families Facing the Future (FFF) is an intervention designed specifically for families with a parent in methadone treatment. FFF is unique because it addresses prevention for children and recovery for parents in a single intervention. The primary goals of the program are to prevent parents’ relapse, help them cope with relapse if it occurs, and teach parenting skills in order to reduce the likelihood of substance use among their children. FFF has been implemented as an adjunct to treatment in several Opioid Treatment Programs, but has not been widely adopted due to various implementation barriers. The aims of this study, therefore, were to (1) assess the perceived feasibility of implementing FFF and (2) identify/describe barriers to implementing FFF. An online survey was used to assess implementation feasibility, while individual qualitative interviews were conducted to explore specific barriers to implementation. Data collection from a total of 40 participants (20 patients and 20 providers) was conducted from August 2022 to October 2022 at two Opioid Treatment Programs in Florida. Analyses revealed high feasibility scores, indicating that FFF was viewed by both patients and providers as a practical intervention to implement. Despite strong perceived feasibility of the intervention, qualitative findings identified several implementation barriers with respect to difficulty attending parent training sessions, aversion to in-home visits, and lack of funding (inability to provide patient incentives/bill insurance). This study provides evidence that while patients and providers view FFF as having high feasibility, significant implementation barriers exist. This paper fills a void in the literature by informing if and which modifications might be necessary to facilitate wider adoption of FFF in real-world Opioid Treatment Program settings.

## Introduction

Families with a parent receiving methadone in Opioid Treatment Program (OTP) settings are characterized by numerous risk factors for substance use.^1,2^ These families often face difficult life circumstances, such as criminal justice involvement, housing instability, unemployment, and poor physical and mental health.^3–5^ These factors place parents in methadone treatment at high risk for relapse and their children at elevated risk for future substance use.^6,7^ Because families with a parent in methadone treatment are at such high risk for substance use and other psychosocial problems, family-based interventions aimed at preventing these issues are needed.^8^ Unfortunately, prevention interventions designed specifically for patients in methadone treatment and their families are rare.^9^

One such program, however, is Families Facing the Future (FFF).^10^ FFF is an established, family-based intervention recognized by the Department of Health and Human Services as a research-supported practice.^11^ The program is for men and women who are parents of young children and are in methadone treatment for opioid use disorder (OUD). FFF wholistically addresses the adverse experiences of having a parent with OUD. FFF is unique in that it was designed specifically for families with a parent receiving methadone for OUD and addresses prevention for children and recovery for parents in the context of a single intervention. The overarching goals of the intervention are to prevent parents’ relapse, help them cope with relapse if it occurs, and teach parenting skills in order to reduce the likelihood of substance use among their children. FFF uses group parent training sessions combined with individual case management services to reduce family-related risk factors (e.g., family dysfunction) and enhance protective factors (e.g., family bonding) related to drug use. FFF has been implemented as an adjunct to treatment in several OTPs,^12^ but has not been widely adopted, presumably due to implementation barriers.

**Figure 1:**
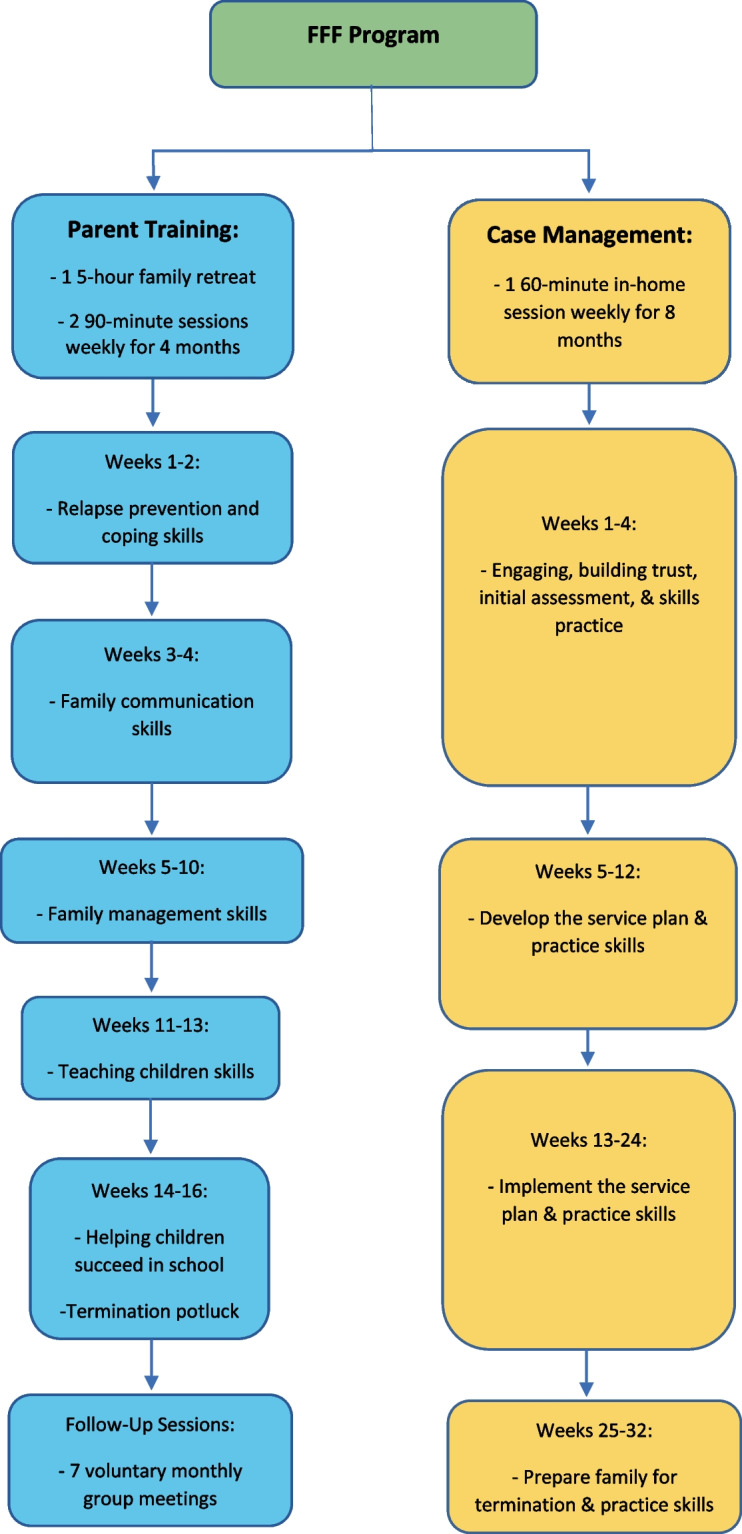
Conceptual diagram

FFF is an intensive, 9-month program. First, families attend a one-time 5-h group retreat at the start of FFF to set goals and establish trust/rapport within the group.^13^ Then, the parent(s) attends 90-min outpatient group sessions twice a week for 16 weeks in an OTP setting (months 1–4). Parent training sessions are designed to teach relapse prevention/coping skills and how to manage their families better.^13^ The optimal group sizes are between 6 and 8 families. Children participate in 11 of the 32 group sessions, as well as the 5-h retreat. Families also receive approximately 2 h of weekly in-home case management to connect them with available services and identify ways to reinforce use of newly learned parenting skills covered in group sessions (months 1–9).^10^ The program is typically delivered by facilitators who have attended a 3-day training and have a background in addiction and working with families. The overall structure of FFF and sequence of services are summarized in Fig. 1.

In previous studies, FFF has been found to reduce parents’ drug use, as well as increase their relapse prevention skills, self-efficacy, parenting skills, and reduce domestic conflict.^14,15^ FFF has also been shown to reduce childhood risk of future drug use (at a trend level significance) and a variety of other problem behaviors.^16^ Despite FFF’s effectiveness, uptake among methadone treatment providers has been low due to a number of implementation barriers that exist. It is possible that the intensity of the program, in terms of both its duration (i.e., 9 months) and mode of delivery (i.e., face-to-face, in-home), may make implementing FFF difficult in real-world OTP settings. For example, patients in methadone treatment often have to rely on public or unreliable transportation, which can make frequent visits to the clinic challenging.^17^ Patients receiving methadone also tend to experience high rates of poverty, unemployment, and mental health problems, which can lead to low/inconsistent patient attendance and engagement.^18–20^

Despite anecdotal reports of a number of common implementation barriers precluding more widespread adoption, no previous studies have empirically examined implementation barriers for FFF. Because of this, researchers are left to speculate regarding which barriers are most important in preventing FFF’s uptake in OTP settings. Wider implementation of FFF has the potential to address key aspects of the escalating opioid overdose crisis given that the program addresses intergenerational addiction, a major driver of opioid addiction and overdose among young people.^21–23^ Few interventions address drug prevention in a treatment context or harness the power of recovering parents serving as prevention agents for their own children. Training parents during their own treatment to act as prevention agents for their children holds promise for intervening and breaking the cycle of addiction.^10^ Interventions, such as FFF, that help keep patients focused on recovery can be powerful treatment adjuncts and may improve recovery outcomes.^24^ While the primary goal of FFF is to prevent illicit drug use, families are also likely to experience a number of additional psychosocial benefits such as reductions in family dysfunction, negative emotional states, school dropout, and delinquency.^12,14,16^ Given the importance of parental drug use as a risk factor for children, it is essential that more parents be targeted during treatment with prevention interventions such as FFF.^25,26^

### The current study

This current study responds to the need for additional data on specific barriers to implementing FFF in OTP settings. This study attempts to fill this gap by presenting data collected directly from patients and providers at OTPs. Engaging the voices of both these groups can help create a better understanding of the most important barriers to implementation from potential consumers of FFF, as well as those responsible for delivering the intervention. A better understanding of these barriers could facilitate greater uptake of FFF across OTPs. The aims of this study were to (1) assess the perceived feasibility of implementing FFF and (2) identify/describe perceived barriers to implementing FFF (as identified by patients and providers). An online survey was used to assess implementation feasibility, while individual qualitative interviews were conducted to explore barriers to implementation. The rationale for a mixed-methods approach is that implementation barriers can be complex and situational, which lends itself to being studied qualitatively.^27^ As such, including both survey and interview data allows for a fuller understanding than would be possible using a survey alone.^28^

## Methods

Study data were drawn from a larger program evaluation of FFF, funded by the Foundation for Opioid Response Efforts. Data collection from 40 participants (20 patients and 20 providers) was conducted from August 2022 to October 2022 at two OTPs in Florida. To be eligible for the study, patients (*n* = 20) had to be at least 18 years old, currently receiving methadone for OUD, and have at least 50% parental custody of a child between the ages of 5 and 17 years old. For providers (*n* = 20), the eligibility criteria were to be at least 18 years old and a current behavioral health provider (e.g., counselor, social worker, certified addiction professional) employed at an OTP. Participants were recruited for diversity across the following parameters: gender, race/ethnicity, and age. This project used an explanatory sequential mixed-methods framework that involved a survey followed by qualitative interviews.^29^ This framework involved first collecting/analyzing quantitative data, and then collecting/analyzing qualitative data to contextualize quantitative findings. The study activities described herein were reviewed and received a “not human subjects research” designation from the Institutional Review Board at University of South Florida. While this study did involve human subjects, it was deemed “program evaluation” (not research) because the data were collected for program improvement purposes.

### Recruitment

Purposive sampling strategies were used to recruit participants. Patients were recruited via posting flyers and handing out cards with study information at two OTPs. Additionally, in partnership with a multi-site addiction treatment system, counselors at two OTPs also distributed study information to patients. Chain referral was also used, whereby each patient who completed the study could refer other potentially eligible patients. Patients were not paid for referrals. Providers were recruited by advertising the study at several staff meetings at the OTP. Recruitment materials explained that the study was part of a larger program evaluation of a family-based intervention for patients in methadone treatment, and interested persons were instructed to call a member of the study team for eligibility screening.

### Data collection

Once eligibility was confirmed, a member of the study team scheduled an appointment at the treatment clinic to conduct the in-person interview. At the beginning of the interview, participants were provided with an informed consent form to read and sign. Participants were assured that their participation was strictly confidential, and they could stop participating at any time. After the consent form was signed, each participant was shown two brief videos introducing the FFF program. The first video (5 min long) provided an overview of FFF’s goals, structure, length, and topics/activities, while the second video (6 min) showed snippets of a real family going through the actual program and providing testimonials of their experiences in FFF. To reinforce the information in the videos, the interviewer spent approximately 5 min providing further detail about the program and answering any participant questions.

Participants then completed a brief 5-min online survey in Qualtrics, answering basic demographic questions (e.g., age, race, ethnicity, gender, educational attainment, marital status, number of children) and a 4-item measure of FFF’s feasibility on a tablet provided by the study team. Measures from the demographic subsections of the National Survey on Drug Use and Health were used.^30^ Psychometric properties of these demographic measures have been well-established in other studies.^31,32^ In addition, the 4-item instrument known as the Feasibility of Intervention Measure was used to assess perceived intervention feasibility.^33^ Each item (FFF seems implementable, FFF seems possible, FFF seems doable, FFF seems easy-to-use) was measured on a 5-point Likert-type scale as follows: 1 = Completely Disagree, 2 = Disagree, 3 = Neither Agree nor Disagree, 4 = Agree, 5 = Completely Agree. The Feasibility of Intervention Measure has demonstrated strong psychometric properties in previous studies.^33^

Following completion of the online survey, participants completed a qualitative interview designed to capture more detailed feedback regarding FFF’s feasibility and perceived barriers to implementation. As part of the qualitative interview, the participants were asked open-ended questions about their perspectives on FFF (e.g., How doable does completing FFF seem to you? Can you describe any challenges to being able to successfully complete/implement FFF? What aspects of FFF did you find most/least appealing?). These questions were chosen on the basis that they were neutrally and clearly worded and conversational in nature. The goal was for the interview to approximate a casual conversation where the participant is the primary speaker. The interview was semi-structured in that specific questions were asked of each participant, but each interview was allowed to take its own course and unfold naturally as new ideas were raised.^34^ Participants received a $50 Amazon e-gift card for completing the interview. Interviews typically lasted about 30–40 min and were conducted at the OTP in a private office space by a trained member of the study team.

### Analyses

For the survey data, descriptive statistics were calculated, whereby percentages are reported for categorical variables and means for continuous variables. Statistical analysis was conducted using Qualtrics software, an online survey, and data analysis program.^35^ For the qualitative portion, each interview was audio-recorded using a digital voice recorder. The audio file was then transcribed verbatim. Qualitative analysis was conducted by four members of the study team using applied thematic analysis techniques in teams of two.^36^ Two team members coded all 20 patient transcripts while the other two members coded all 20 provider transcripts. NVivo (version 12) was used to code the interview data.^37^

First, each team member read each transcript twice to become familiar with the data. On the second reading, initial ideas for coding were noted and discussed by the team. Next, all relevant data related to each coding category was systematically coded and collated across the entire dataset. Then, initial codes were sorted into potential themes, and all text data relevant to each initial theme were gathered. After this, each team reviewed and refined the devised set of initial themes by checking if the data cohered together meaningfully within each theme. The specifics of each theme were then decided upon, and the overall story of the data emerged. In the final step, the report was written, and compelling excerpts from participants were selected to illustrate each theme. After 30 interviews, it was determined that saturation was achieved as no new patterns emerged from the data. Themes related to implementation barriers were the focus of the qualitative analysis.

## Results

### Sample characteristics and feasibility results

Demographic characteristics for the sample, stratified by study group, are displayed in Table 1. The sample consisted of 40 participants (20 patients; 20 providers) of whom nearly two-thirds were female. The mean age of the total sample was 40.1 years, and the average number of children for each participant was 1.7. Just over two-thirds were unmarried, and the vast majority (92.5%) were employed full time. Regarding race, 65% identified as White, 17.5% as Black, 5% as Asian/Pacific Islander, 5% as bi/multiracial, and 7.5% as “other.” Fifteen percent identified as Hispanic. Nearly two-thirds of the total sample reported having more than a high school diploma. Additionally, the mean scores on all four items of the Feasibility of Intervention Measure were as follows (out of 5): (1) FFF seems implementable = 4.3, (2) FFF seems possible = 4.5, (3) FFF seems doable = 4.6, (4) FFF seems easy to use = 4.2. The mean score across the entire sample was 4.3 out of 5, with patients having a mean score of 4.5 out of 5, and providers a mean score of 4.2 out of 5.

**Table 1:** Sample characteristics and feasibility

Measure	Patients (*n* = 20) *n* (%)	Providers (*n* = 20) *n* (%)	Total (*n* = 40) *n* (%)
Age (mean)	37.3	42.9	40.1
Number of children (mean)	2.4	0.9	1.7
Gender			
Female	15 (75.0)	11 (55.0)	26 (65.0)
Race			
White	15 (75.0)	11 (55.0)	26 (65.0)
Black	2 (10.0)	5 (25.0)	7 (17.5)
Asian/Pacific Islander	0 (0.0)	2 (10.0)	2 (5.0)
Bi/multiracial	1 (5.0)	1 (5.0)	2 (5.0)
Other	2 (10.0)	1 (5.0)	3 (7.5)
Ethnicity			
Hispanic	3 (15.0)	3 (15.0)	6 (15.0)
Marital status			
Married	5 (25.0)	8 (40.0)	13 (32.5)
Employment status			
Full-time	17 (85.0)	20 (100.0)	37 (92.5)
Part-time	3 (15.0)	0 (0.0)	3 (7.5)
Education			
Some high school	3 (15.0)	0 (0.0)	3 (7.5)
High school diploma/GED	11 (55.0)	1 (5.0)	12 (30.0)
Some college	4 (20.0)	0 (0.0)	4 (10.0)
Bachelor’s degree	1 (5.0)	10 (50.0)	11 (27.5)
Graduate degree	0 (0.0)	8 (40.0)	8 (20.0)
Other	1 (5.0)	1 (5.0)	2 (5.0)
Feasibility			
FFF seems implementable (mean)	4.4	4.1	4.3
FFF seems possible (mean)	4.4	4.6	4.5
FFF seems doable (mean)	4.7	4.4	4.6
FFF seems easy to use (mean)	4.5	4.0	4.2

### Qualitative results on barriers to implementation

#### Attending parent training sessions

An aspect of FFF that was identified as a barrier by both patients and providers was attending parent training sessions at the OTP. These sessions, held twice a week for 90 min each, were largely viewed as onerous given that patients would have to take 3 h (not including the commute to and from the clinic) out of their busy week to attend. Attendance at two weekly sessions of this length would cause patients to have to take time off from their jobs or significantly rearrange their work schedules. Additionally, childcare was seen as a barrier to attending these sessions. Attending these sessions meant that some patients would not only have the inconvenience of finding childcare twice a week, but also incur the expense. Additionally, these sessions required patients to make extra trips to the OTP, which patients viewed as burdensome and providers viewed as unrealistic. Most patients relied on some form of public (e.g., bus) or unreliable transportation (e.g., asking a friend) which made regular attendance at these sessions appear daunting. This participant articulates her transportation difficulties, when asked if she could attend two 90-min sessions a week at the OTP:

So right now, I have none (transportation), I’d have to try and find a way to get there (to the OTP). And I really definitely wouldn't be able to pay (for an Uber). I wouldn’t be able to take $20 of my grocery money and use it on a ride. Maybe I could get transportation through a Medicaid cab. Medicaid might be able to cover that. I don’t know.

It is also worth noting that in the current version of FFF, children are required to attend several group sessions with the parent(s). Some patients and providers saw this as a barrier. One of the reasons was that 90 min was viewed as too long to ask a child to “pay attention” and “remain focused,” especially for young children. The possibility of older teenagers being in a group with preadolescent children was also raised as a potential problem. For example, older adolescents may be more likely to use language or discuss topics/problems that may be developmentally inappropriate for younger children. Also, the requirement to involve children in selected group sessions meant that children may have to miss school unless sessions are scheduled on evenings or weekends, which was also viewed as inconvenient. Additionally, some parents were reluctant to bring their children to the OTP. They either did not want their child to know they were in treatment or were reluctant to expose them to the addiction treatment environment. The following patient expresses reluctance about bringing her children to the OTP:

The thing I would be iffy about is bringing my kids to a methadone clinic…My kids have never seen me high…They’ve never seen any kind of drugs. I try to shelter them from that world. I’ve even been hearing some patients get into fights. I just don't want my kids around any of that.

#### Aversion to in-home visits

The in-home component was another commonly raised issue by both patients and providers. Patients were generally averse to having someone visit their homes because it felt “intrusive.” Most were averse to home visits out of shame over their current living situation. In fact, it was not uncommon for patients to report recently living in a homeless shelter, “on the streets,” or “couch surfing.” Patients expressed reservations over having someone visit their home environment when experiencing such periods of housing instability or homelessness. Even for patients in more stable living situations (e.g., own or rent their home), home visits were seen as a tolerated aspect of the program, but not a welcomed one. This patient discusses her discomfort with in-home visits:

Maybe some people wouldn’t mind a provider coming in to their home, but me personally, I don’t want anyone to come into my house. Home is sacred. Home is somewhere that’s supposed to be private from everybody. Not saying I’m hiding anything, but I just wouldn’t want anyone to come into my home.

The issues for providers largely revolved around the logistics of in-home visits. For example, home visits can be potentially dangerous for providers, leaving them vulnerable to certain safety hazards (e.g., driving to what some providers described as “high-crime areas”), which was another perceived barrier to implementation. Concerns regarding the time-consuming nature of home visits were also voiced. Because providers tended to have large caseloads, time was seen as a valuable commodity that was not best spent driving to various patient homes. Traveling to patients’ homes every week also raises issues around mileage reimbursement and wear-and-tear on providers’ personal vehicles. The following provider articulates their overall aversion to in-home visits:

We decided to stop in-home programs because we have to drive there and drive back home. And you got to pay that staff for the time. So, all in-home stopped…My thoughts for getting this off the ground would be to get rid of the home-based part because that's just a whole lot to figure out.

#### Lack of funding (Inability to provide patient incentives/bill insurance)

Some participants lamented the fact that patients were not paid or incentivized to participate in the program. It was believed that the lack of incentives would lead to difficulties recruiting and retaining patients in the program, especially considering that FFF was seen as an adjunctive intervention to traditional methadone treatment. The prevailing sentiment was that there would be high patient attrition if they did not receive a tangible (ideally financial) reason to participate in the program. Financial remuneration was viewed as necessary because participation in the program was viewed as a net negative without it. That is, although their recovery and family functioning would benefit from the curriculum, participation comes at a cost (e.g., significant time commitment [8-month program, 4 h a week], hassle/cost of finding childcare, transportation burden of extra trips to OTP, enduring imposition of in-home visits). The need for patient incentives was seen as a way to offset the “hidden” costs of participation, as this provider explains:

You have to have incentives. I know people might be thinking that they shouldn’t need incentives to do it, but it really is a huge part…I think financial incentives would be ideal. Money makes the world go round. But just helping patients out with things like coupons, transportation, housing, or helping them out with being able to get items that they need.

Providers also believed that OTPs would not be able to bill insurance (namely Medicaid/Medicare) for delivering the program. Considering that OTPs often struggle with underfunding, the inability to bill insurance for the time providers spend on program delivery was important. Time spent delivering the program meant less time available for billable services, including assessments or individual/group therapy. The inability to bill for program delivery was exacerbated by the program’s length. The inability to bill made implementing the program seems less sustainable, as this provider states:

I mean billing for services is very specific. We’re mostly just billing for dosing and individual treatment sessions that folks are getting. We don’t bill Medicaid for any of our family services. It’s almost like, well, we can’t bill, so we don’t. I don’t see how we would get these services covered by insurance companies or Medicaid or Medicare.

## Discussion

Although FFF has been studied extensively over the last two decades,^10,13–16^ the program has not been widely adopted and implemented by OTPs. It is believed that this has been largely due to significant barriers to implementation. To date, no previous studies have empirically examined barriers to implementing FFF, and additional data on this topic are needed. These findings add to our limited understanding of the program’s feasibility and factors that may be contributing to the low uptake across OTPs. Studies that can inform wider implementation of FFF are especially important because this particular evidence-based program targets families with a parent in methadone treatment, a high-risk group for a variety of psychosocial problems.^1–7^

Study results indicate that FFF was viewed by patients and providers alike as a useful intervention to implement. As indicated by scores on the Feasibility of Intervention Measure (mean score of 4.2 out of 5), the program was seen as highly implementable, possible, doable, and easy-to-use. Taken together, these scores showed a high level of feasibility across the sample, with generally consistent findings across patients and providers.

Qualitative findings, however, did uncover some perceived barriers to implementation. The first of which was attending numerous group parent training sessions at the OTP. Attending two 90-min sessions every week over the course of 4 months was viewed as onerous due to having to find childcare, incompatibility with work schedules, having to make extra in-person visits to the OTP, and the inconvenience of bringing their child to some of the group sessions. This finding has implications for patient recruitment and retention. Patients may be less likely to participate and more likely to prematurely dropout if they view these aspects as undesirable. Additionally, OTPs may decide not to adopt FFF over concerns with these issues. A few program adaptations could be considered in light of this finding. Shortening each in-person group session (e.g., from 90 to 60 min) and/or reducing the frequency of twice weekly sessions to a single session each week might seem less daunting and may be a logical next step in any planned adaptations to make FFF more appealing to outpatient methadone treatment settings. Additionally, OTPs could offer onsite childcare to make it easier for patients who are unable to find/afford childcare to attend. Delivering the parent training sessions (partly or fully) in a virtual format (instead of in-person at the OTP) might also serve as an implementation strategy to address this barrier. Conducting support groups and other treatment visits via telehealth have become fairly commonplace since the COVID-19 pandemic and have been shown to be as effective as face-to-face delivery.^38–40^

The in-home component was also identified as a barrier to implementation. The program calls for about 2 h of weekly in-home case management to connect families with available services and identify ways to reinforce use of new parenting skills.^10^ Patients tended to view in-home visits as “intrusive” and expressed discomfort about letting staff into their home environments, which sometimes involved sleeping in shelters, on the streets, or couch surfing. In-home visits can indeed offer an intimate view of the patient’s family life in their natural setting,^41,42^ but recent evidence suggest that behavioral health patients may be increasingly offput by home visits.^43,44^ Providers also had reservations about home visits due to logistical concerns (e.g., safety issues, travel time). This finding suggests that the home visit component may steer patients away from participating in FFF and deter OTPs from implementing the program at their site. Possible program adaptations might include delivering the case management portion of the program remotely through video chats, phone calls, and/or text messages. Recent research indicates that not only is remote case management effective,^45^ but patients might actually prefer it.^46^

Another perceived barrier was the fact that patients were not paid or otherwise incentivized for their participation. It was believed that OTPs would have a difficult time recruiting and retaining patients without incentives to offset “hidden” costs associated with transportation and childcare. Although financial incentives would be most effective, paying patients to participate would, of course, constitute its own implementation barrier for OTPs in the inner context. As an alternative, non-financial incentives might be considered.^47,48^ For example, patients receiving medication who show consistent participation for a pre-determined period of time could be given take-home privileges, whereby patients are given multiple methadone doses at a time, removing the need for daily OTP visits. Earning take-home privileges has a long history of being used as an effective incentive.^49–52^ Incentives such as free parking passes, vouchers for public transportation, and time-limited childcare during clinic visits could also be considered as non-monetary incentives.^10^ In addition, the inability to bill insurance (particularly Medicaid and Medicare) was another perceived barrier in the outer context. While the curriculum is typically delivered by a case manager, OTPs could have a licensed provider facilitate the program. Delivery by a licensed provider may create a pathway for provision of more billable services.

A few methodological issues warrant mention. A strength of this study was the utilization of a mixed-methods approach which allowed for a fuller understanding than would have been possible using a survey alone. However, as this was a nonprobability sample, our results may not be representative of all OTPs, and generalizations should be made prudently. Data for this study were collected across two OTP sites, which also limit the generalizability. Given that face-to face interviewing was used as a means of data collection, interviewer effects and social desirability bias may have been a possibility.

## Implications for Behavioral Health

Despite these limitations, this study has implications for the behavioral health field. These findings add to the limited amount of empirical data on barriers to implementing FFF. The data presented here provide important insights for OTPs as they attempt to implement this intervention at their sites and inform future adaptations to the curriculum. While these findings pertain specifically to FFF, they likely also apply to other similar family-based interventions delivered at OTPs. Our study provides evidence that while FFF is seen as implementable, significant barriers exist. This study directly fills a gap in the literature by being the first study to empirically assess FFF’s feasibility and implementation barriers. It underscores just how powerful the “hidden costs” of interventions can be in deterring patients from participating and OTPs from delivering these programs at their sites. Developers of family-based interventions may wish to consider the issues raised in this paper when creating new programs to be implemented in real-world OTP settings.
